# Host transcriptome profiling for resistance against lumpy skin disease (LSD)

**DOI:** 10.1186/s13104-025-07388-9

**Published:** 2025-07-15

**Authors:** Mohammad Hossein Banabazi, Steven Van Borm, Tomas Klingström, Adnan Niazi, Kris De Clercq, Laurent Mostin, Andy Haegeman, Dirk Jan de Koning

**Affiliations:** 1 Department of Animal Biosciences, Swedish University of Agricultural Sciences, Box 7023, Uppsala, 750 07 Sweden; 2 Sciensano, Rue Juliette Wytsmanstraat 14, Brussels, 1050 Belgium; 3 SLU Bioinformatics Infrastructure, Swedish University of Agricultural Sciences, Box 7023, Uppsala, 750 07 Sweden

**Keywords:** Cattle, Lumpy skin disease (LSD), *IL1RAP* gene, Transcriptome profiling, Host determinants, Differentially expressed genes (DEG)

## Abstract

**Objective:**

Lumpy skin disease (LSD) is an acute or subacute systemic viral disease of cattle that shows variation in the response of cattle to LSD virus infection. To better understand the mechanisms underlying this response diversity in field studies and under carefully controlled artificial infections, we studied the differentially expressed genes (DEGs) between two resilient versus three susceptible Holstein bulls before an infection challenge and three time points after that.

**Results:**

The host transcriptome profiling revealed that IL1RAP gene expression could be a potential determinant in distinguishing between resilient and susceptible cattle (padj < 0.05). It was significantly shifted from up-regulated prior to infection to down-regulated three days post-infection in the LSD-resilient cattle. Its expression remained up-regulated among the susceptible cattle post-infection compared to pre-infection. The results showed that seven days post-infection may be a critical time point for LSD infection. The Gene Ontology (GO) and KEGG (Kyoto Encyclopedia of Genes and Genomes) pathway enrichment test showed a few enriched GO terms and pathways relevant to the LSD and the involvement of the IL1RAP gene. This pilot study, with limited statistical power, is the first to investigate bovine gene expression profiling in response to LSDV.

**Supplementary Information:**

The online version contains supplementary material available at 10.1186/s13104-025-07388-9.

## Introduction

Lumpy skin disease (LSD), an acute or subacute systemic viral disease of cattle, is a major global health threat to livestock [1]. LSD was first diagnosed in 1929 in Zambia and spread into the Middle East in 2012 and Europe in 2015 [2]. Since 2019, LSD recombinant virus strains are spreading in large parts of Asia [3]. There is host variation in the response of cattle to lumpy skin disease virus (LSDV) infection in field studies. Between 20 and 50% of the animals have no clinical signs (asymptomatic) when a herd is infected with LSDV [4]. To better understand the mechanisms underlying the response diversity, we studied the differentially expressed genes (DEGs) between symptomatic and asymptomatic cattle at each time point before and after virus challenge as well as among non-recovered animals over time.

## Materials and methods

Our samples were taken from five Holstein bulls that served as the control animals in a large vaccine trial at Sciensano Belgium [5]. These bulls were purchased by Sciensano at about 6 months of age. They were sampled for whole blood using Tempus™ Blood RNA Tubes (ThermoFisher Scientific) and experimentally infected five days later at Sciensano (Belgium) with LSDV via injection in the vena jugularis and the neck with a LSDV strain derived from Israel [5]. The bulls were given a daily clinical score that takes account of changes in food intake, body temperature, general responsiveness, amount of LSD related nodules, localization of LSD related nodules, swelling at the inoculation site, and prescapular lymph node swelling. Three bulls showed clear LSD symptoms, and two did not. All bulls were also sampled three, seven, and fifteen days post-infection (dpi). Twenty whole RNA samples were isolated using the Tempus™ Spin RNA Isolation Reagent Kit (ThermoFisher Scientific) according to the manufacturer’s instructions. All RNA showed RNA Integrity Number equivalent (RINe) scores above 9.0 (High Sensitivity RNA ScreenTape assay, Agilent Technologies). RNA sequencing was performed through a QuantSeq approach [6] by Illumina NextSeq500 using a 150 HO sequencing kit at the Neuromics Support Facility of VIB University of Antwerp, Center for Molecular Neurology, Belgium. This method results in a single unique fragment per transcript, thus simplifying the quantification of gene expression. After data quality control using FastQC v0.11.8, the 150-bp single reads were trimmed using bbduk.sh script available in BBMAP suite v38.94 [7]. The filtered reads were mapped on the Bos taurus reference genome (ARS-UCD1.2, Ensemble release 105) by HISAT2 aligner [8]. The features included in the Bos taurus annotation (the same release) were counted on the individual assembled transcriptomes by featureCounts v2.0.1 [9]. Raw read counts were normalized and differential gene expression analysis was performed for the asymptomatic versus symptomatic contrasts in each time point as well as for symptomatic animals in each post-infection time point versus pre-infection time using the DESeq2 v1.32.0 package [10] under R v. 4.1.3 [11]. The former contrasts reveal the genes involved in the susceptibility to disease, and the last show which genes would be involved when the infection is induced and evolves to symptomatic LSD. After differential expression analysis, the resulting p-values were adjusted for multiple testing using the Benjamini–Hochberg procedure. DEGs with adjusted p-values (p_adj_) < 0.05 were considered significant. Gene Set Enrichment Analyses were done for gene ontology terms (GO) and KEGG pathways through R packages: clusterProfiler v4.2.2 [12] and org.Bt.eg.db V3.14.0 [13].

## Results

On average 25.6 million reads per sample were generated by sequencing. The raw reads were minimally trimmed about 0.7% of total numbers. The filtered reads were aligned on the reference genome in an average rate of 95.4% (Table S1). One symptomatic sample on three dpi was recognized as an outlier in Principle Component Analysis (PCA) and removed (Figures S1 and S2). This sample had the lowest RNA concentration. The alignment rate for all samples was 95,4% on average. The asymptomatic vs. symptomatic contrasts revealed that 20, 34, 364, and 37 genes were significantly differentially expressed (p_adj_ < 0.05) five days prior to (pre-infection), three, seven, and fifteen days post-infection (dpi), respectively (Table S2, and Figures S3-A). The limited number of differentially expressed genes is clearly reflected in Figure S1 where there is no clear clustering of asymptomatic and symptomatic animals.

Differentially expressed genes (DEGs) in the pre-infection time point reveal good candidate determinants for susceptibility to LSD. They are expressed without the infection being present but may be predictors for disease outcome. The experimental infection may influence in the gene expression and activate GO and pathways that do not necessarily result from the induced infection and propose confounding determinants. The symptomatic vs. asymptomatic contrast on pre-infection revealed a few DEGs with an interesting trend. For example, Interleukin 1 Receptor Accessory Protein (IL1RAP) gene was significantly down-regulated five days pre-infection (p_adj_ < 0.05) in cattle that showed the symptoms after challenge (susceptible) but was up-regulated three dpi in the same animals. This gene is located on chromosome one and includes twelve exons ordered as four transcripts. IL1RAP is an essential regulator of redox homeostasis and a cell-surface protein best known as a co-receptor for interleukin 1 receptor signaling [14].

Also, its expression was significantly up-regulated after challenge over time among symptomatic animals (p_adj_ < 0.05). IL1RAP was thus differentially expressed at each time point post-infection versus pre-infection (Table 1). It can be concluded that IL1RAP may have a key role in LSD susceptibility and the control of the disease where high expression of IL1RAP is associated with disease occurrence.

**Table 1:** Gene expression of *IL1RAP* gene (*ENSBTAG00000013205*) in different contrasts (p_adj_ < 0.05)

Contrast		log_2_ Fold Change	pvalue	padj
**Symp vs. Asymp** (BaseMean = 21.432)	**pre-infection**	-20.158	6.55E-11	2.28E-07
	**3 dpi**	19.558	1.28E-08	3.81E-05
	**7 dpi**	0.389	0.89	0.99
	**15 dpi**	-0.990	0.73	1
**Symptomatic over time** (Post-infection vs. pre-infection) (BaseMean = 21.528)	**3dpi vs. pre-infection**	14.998	8.25E-07	0.0022
	**7dpi vs. pre-infection**	17.296	1.19E-08	2.08E-05
	**15dpi vs. pre-infection**	15.721	2.27E-07	0.00034

Day seven post infection showed the highest number of significant DEGs (p_adj_ < 0.05) and the relatively low number of shared ones with other time points (Table S2, Table S3, Figure S3, Figure S4). In addition, the enriched GO terms were only seen in symptomatic animals vs. pre-infection ones on 7dpi (Figure S5). They were mostly relevant to immune responses. These results indicate that day 7 is critical in LSD infection.

Differential expression of *IL1RAP* show subsequent enrichment for the cytokine-cytokine receptor interaction pathway with five other DEGs (p_adj_ < 0.05) between symptomatic animals on seven dpi compared to pre-infection time (Figure S5). The therapeutic potential of targeting interleukin-1 family cytokines has been suggested in chronic human inflammatory skin diseases that show similar symptoms to LSD [15]. It was suggested as promising target for immunotherapy of metastasis [16] using antibodies in humans [17].

The expression of *IL1RAP* prior to any LSD outbreak may promise to distinguish the resilient and susceptible cattle for LSD through a high-resolution and low false discovery rate (FDR) diagnostic Real-time PCR test. Some human studies have already shown its involvement in similar diseases.

## Limitations

The work presented here was a retrospective pilot study to investigate the utility of bio-banked whole blood samples to analyze the transcriptome of cattle at different time points before and after artificial infection with LSDV. While the study clearly showed the merits of routine biobanking for retrospective analyses it also has clear limitations: (1) the number of samples is limited by the experiment that was previously done. Transcriptomics was not the main purpose of the original experiment. (2) the time-points of sampling are ad-hoc and may not reflect the key events in terms of gene expression. Other time points may have given a more informative gene expression signature. The main weakness of the present study is that we only had five biological replicates, with three, respectively two replicates per disease outcome. This means that any statistical inferences from the study are preliminary and require replication in a larger study. Despite the limited sample size, the results make biological sense. Another limitation of the present study was the use of a fragment based sequencing approach as opposed to whole-transcriptome sequencing. This is more cost-effective but prevents the identification of splice variation or transcript length variation between animals. Given the cost and severity of animal experiments involving potentially lethal viruses, transcriptome studies will always have to be planned as a complement to more invasive studies like transmission experiments or vaccine trials. This implies a key role for systematic biobanking to allow the accumulation of sufficient suitable samples across studies. In turn, this requires meticulous data recording and documentation of metadata.

## Electronic supplementary material

Below is the link to the electronic supplementary material.

Supplementary Material 1: Table S1 (Additional Excel File 1). Overall statistics for every sample regarding raw number of reads, amount of trimming, number of clean reads and mapping rate.

Supplementary Material 2: Table S2 (Additional Excel File 2). The significant Differentially Expressed Genes (DEGs) between symptomatic and asymptomatic cattle at five days pre-infection, three, seven and fifteen days post-infection time points (padj < 0.05).

Supplementary Material 3: Table S3 (Additional Excel File 3). The significant Differentially Expressed Genes (DEGs) among symptomatic cattle over time post-infection vs. pre-infection (padj < 0.05).

Supplementary Material 4: Figure S1. PCA plots of all samples (A), and without the outlier sample (B). R31 and R32 are the two asymptotic animals.

Supplementary Material 5: Figure S2. Heatmap of the distribution of all samples (A), and without the outlier simple (B). The color-coded values represent clustering distances where darker colors indicate shorter clustering distance between samples.

Supplementary Material 6: Figure S3. MA-Plots of the statistically significant Differentially Expressed Genes (DEGs), indicated by red dots, between symptomatic and asymptomatic animals (padj < 0,05) five days pre-infection, three, seven and fifteen days post-infection (A), and among symptomatic cattle (padj < 0.05) over time (B). The X-Axis represents the means of normalized counts. The horizontal blue lines indicate the cut-off for 1.4 fold-change in expression.

Supplementary Material 7: Figure S4. Venn diagram of the shared numbers of the Significant Differentially Expressed Genes (SDEGs) between symptomatic vs. asymptomatic animals five days pre-infection, three, seven and fifteen days post-infection (A), and between symptomatic cattle overtime (B).

Supplementary Material 8: Figure S5. Gene Ontology (GO) enrichment test (padj < 0,05) for the symptomatic vs. asymptomatic contrasts (A) and symptomatic cattle over time (B).

## Data Availability

Raw RNA Sequencing reads are publicly available in the EBI ArrayExpress repository (https://www.ebi.ac.uk/biostudies/arrayexpress/) with accession number E-MTAB-12547. The blood samples used in this study were finished completely during RNA extractions and are no longer available.

## Abbreviations

DPI: Days post infection
DEG: Differentially Expressed Genes
GO: Gene Ontology
IL1RAP: Interleukin 1 Receptor Accessory Protein
KEGG: Kyoto Encyclopedia of Genes and Genomes
LSD: Lumpy Skin Disease
LSDV: Lumpy Skin Disease Virus
PCA: Principle Component Analysis
RINe: RNA Integrity Number equivalent
