# Supplementary figures and images for: Host transcriptome profiling for resistance against lumpy skin disease (LSD)
Source: BMC Res Notes. 2025 Jul 15;18:299. doi: 10.1186/s13104-025-07388-9 (PMC12265363; doi:10.1186/s13104-025-07388-9)

**A**

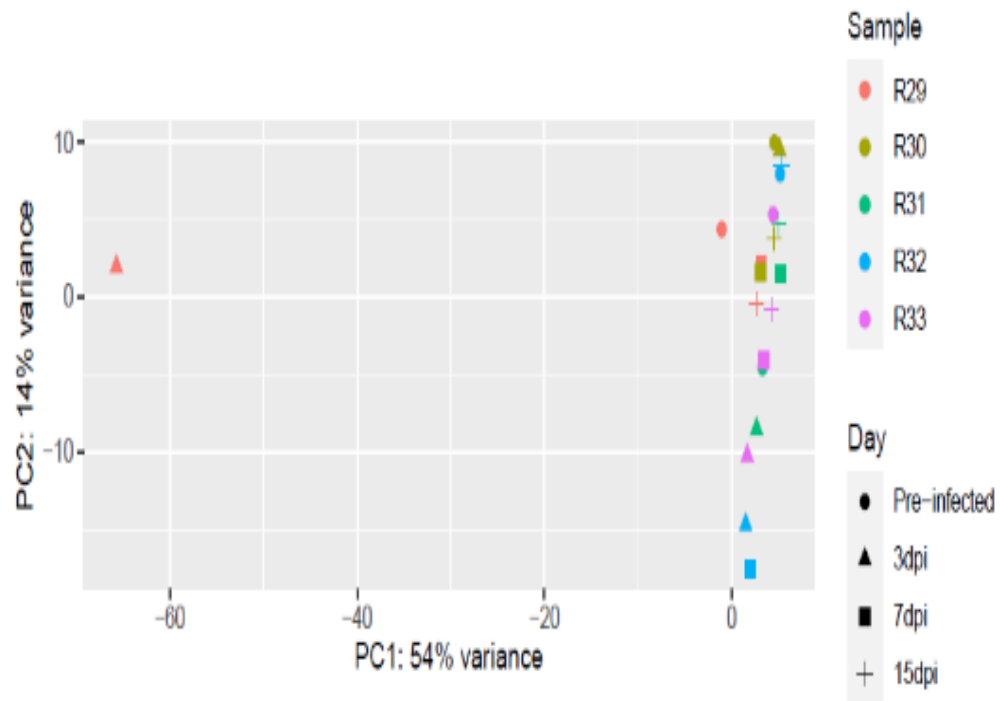

**B**

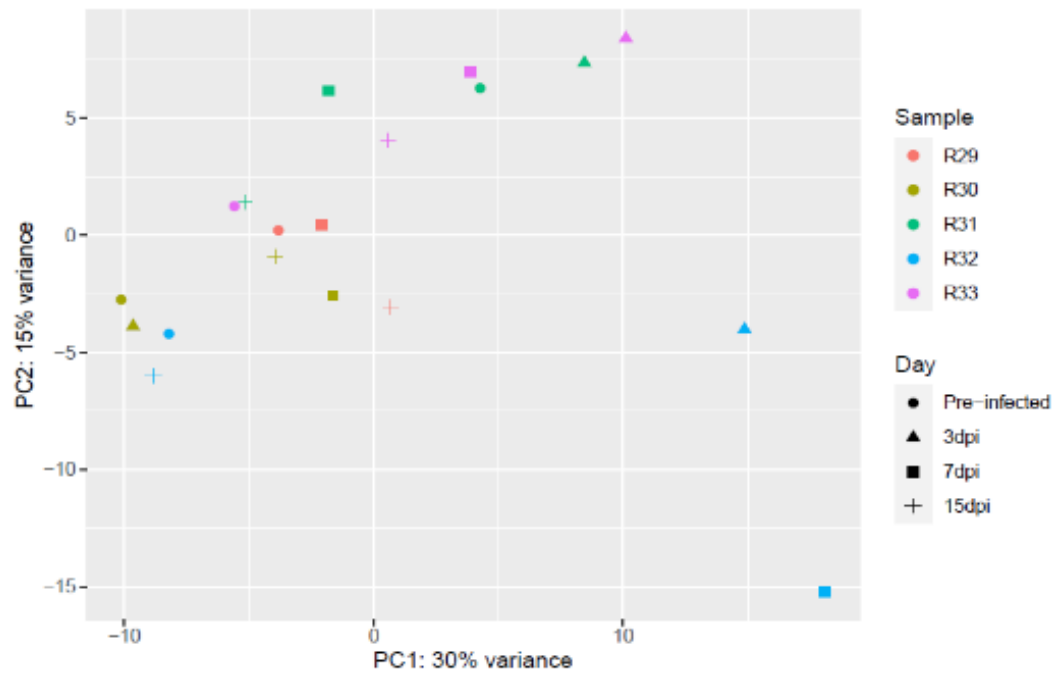

Supplement: Supplementary file 4 — Supplementary Material 4: Figure S1. PCA plots of all samples (A), and without the outlier sample (B). R31 and R32 are the two asymptotic animals. [file 13104_2025_7388_MOESM4_ESM.pdf]

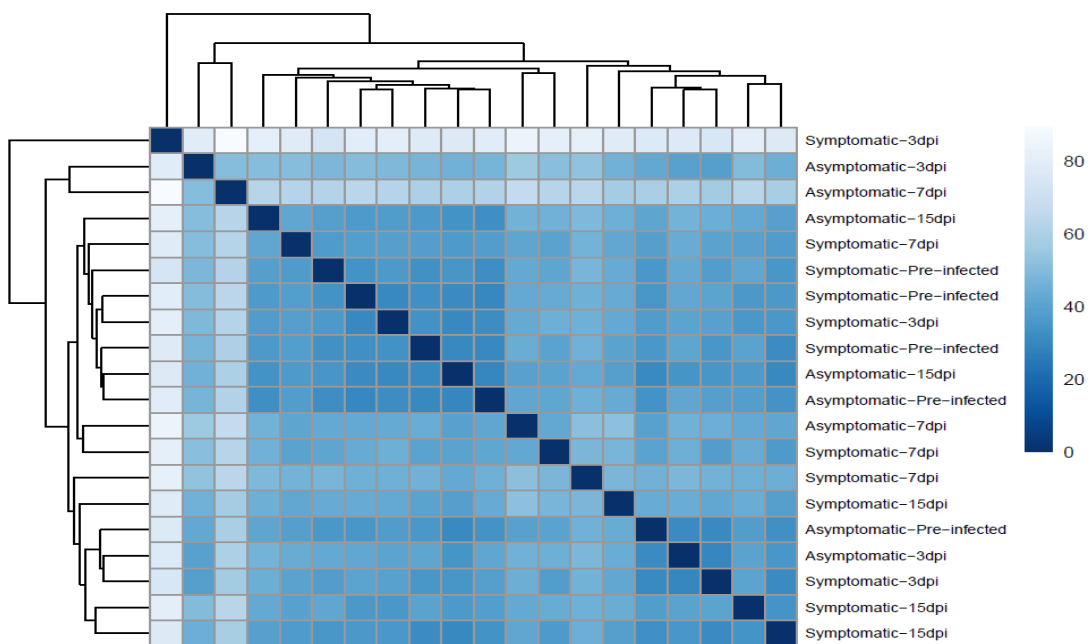

A

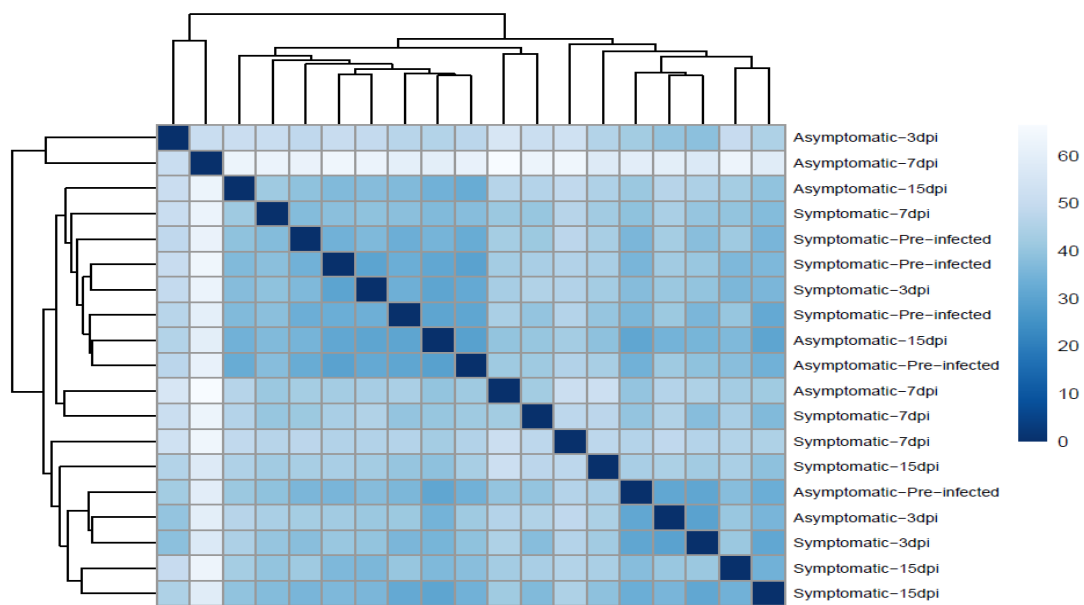

B

Supplement: Supplementary file 5 — Supplementary Material 5: Figure S2. Heatmap of the distribution of all samples (A), and without the outlier simple (B). The color-coded values represent clustering distances where darker colors indicate shorter clustering distance between samples. [file 13104_2025_7388_MOESM5_ESM.pdf]

**A**

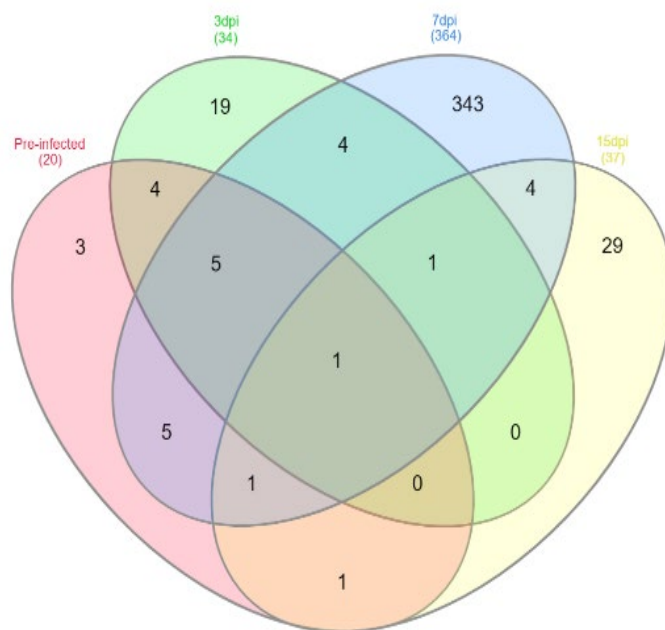

**B**

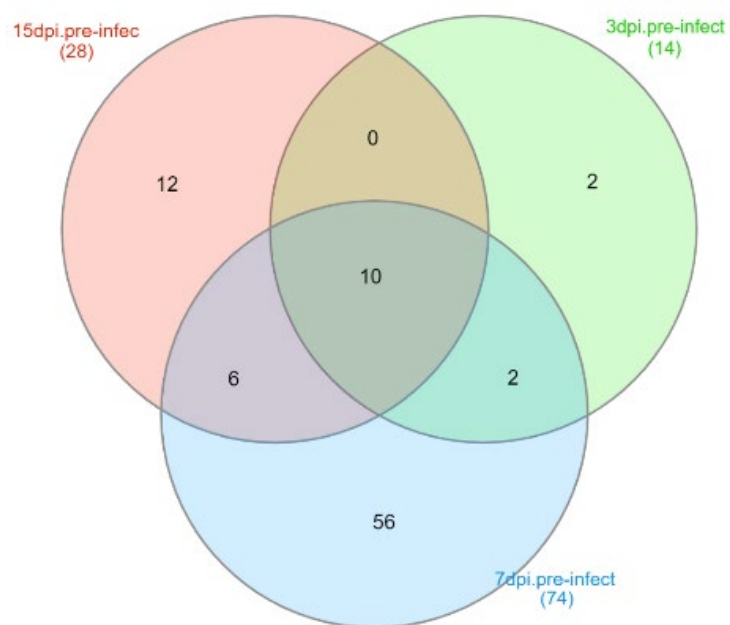

Supplement: Supplementary file 7 — Supplementary Material 7: Figure S4. Venn diagram of the shared numbers of the Significant Differentially Expressed Genes (SDEGs) between symptomatic vs. asymptomatic animals five days pre-infection, three, seven and fifteen days post-infection (A), and between symptomatic cattle overtime (B). [file 13104_2025_7388_MOESM7_ESM.pdf]

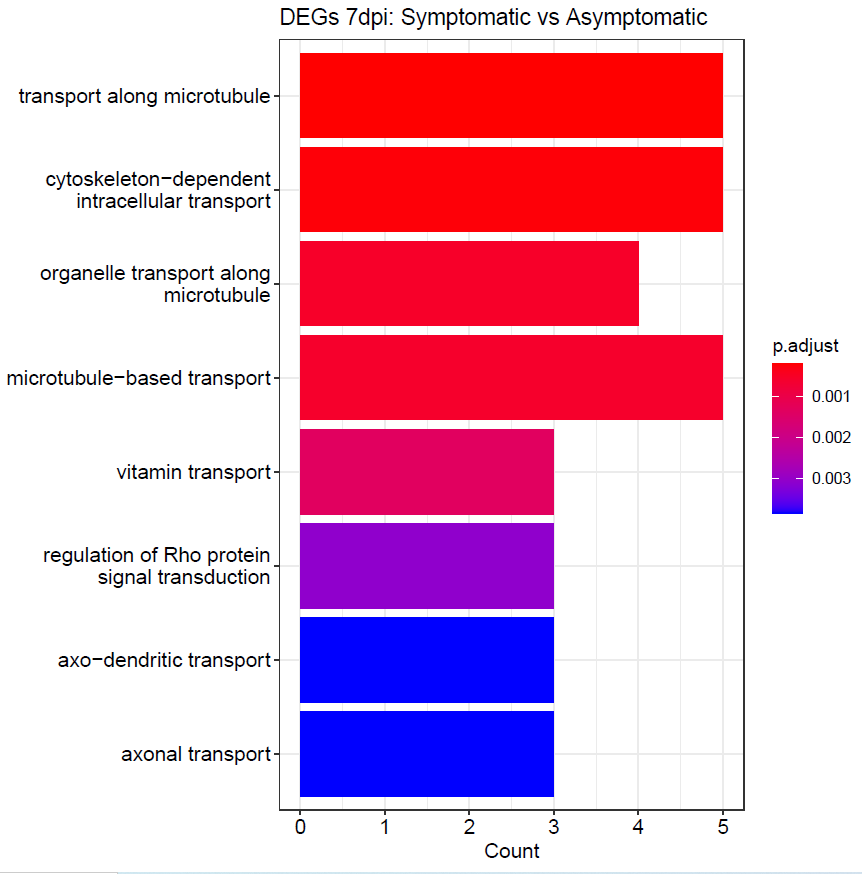
**
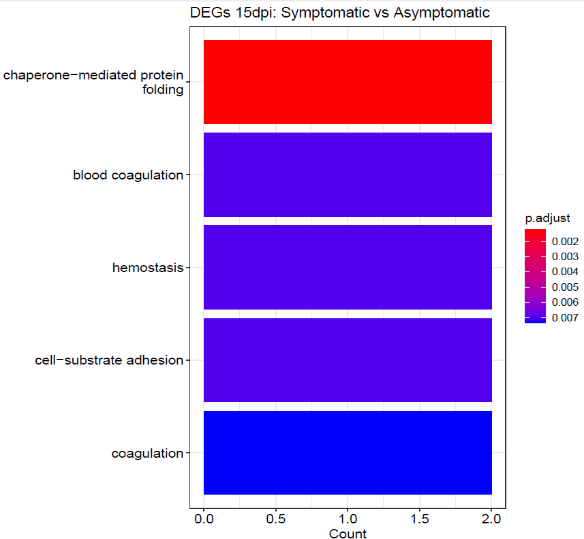

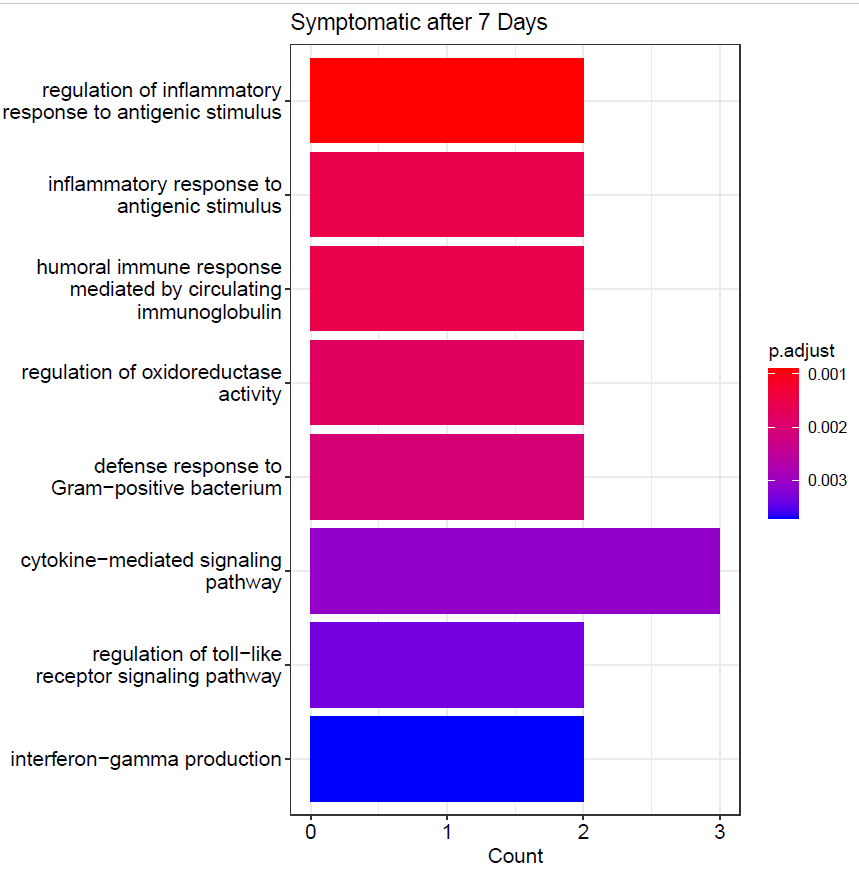
**
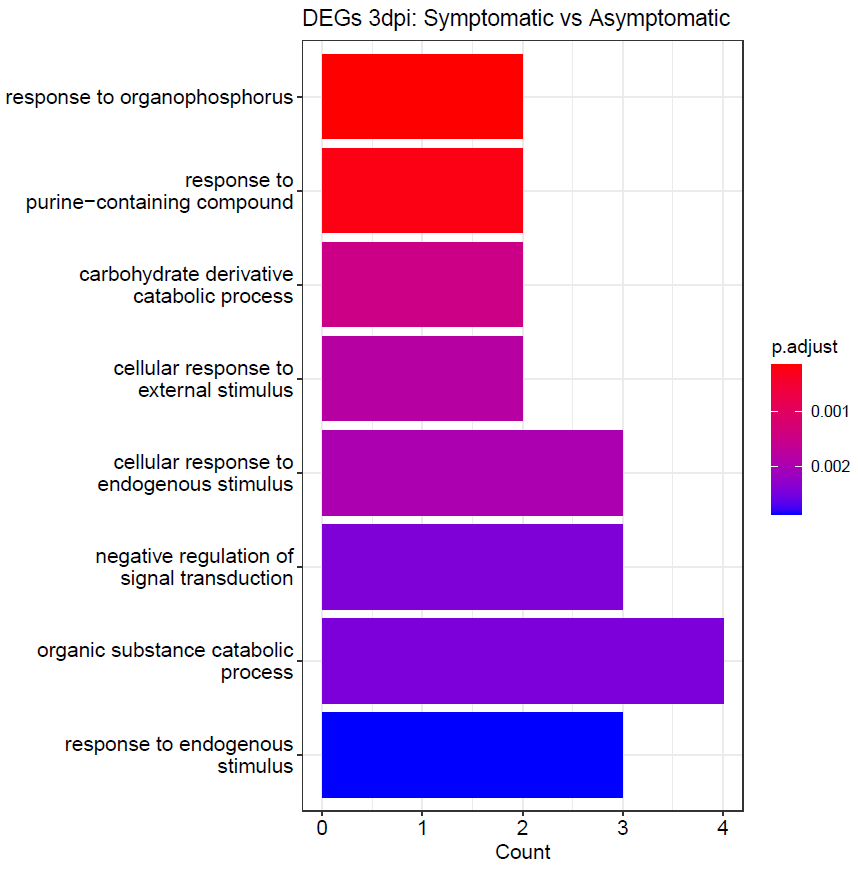


**A**

**B**

Supplement: Supplementary file 8 — Supplementary Material 8: Figure S5. Gene Ontology (GO) enrichment test (padj < 0,05) for the symptomatic vs. asymptomatic contrasts (A) and symptomatic cattle over time (B). [file 13104_2025_7388_MOESM8_ESM.docx]
