# Supplementary material for: Host transcriptome profiling for resistance against lumpy skin disease (LSD)
Source: BMC Res Notes. 2025 Jul 15;18:299. doi: 10.1186/s13104-025-07388-9 (PMC12265363; doi:10.1186/s13104-025-07388-9)
Supplement: Supplementary file 6 — Supplementary Material 6: Figure S3. MA-Plots of the statistically significant Differentially Expressed Genes (DEGs), indicated by red dots, between symptomatic and asymptomatic animals (padj < 0,05) five days pre-infection, three, seven and fifteen days post-infection (A), and among symptomatic cattle (padj < 0.05) over time (B). The X-Axis represents the means of normalized counts. The horizontal blue lines indicate the cut-off for 1.4 fold-change in expression. [file 13104_2025_7388_MOESM6_ESM.pdf]

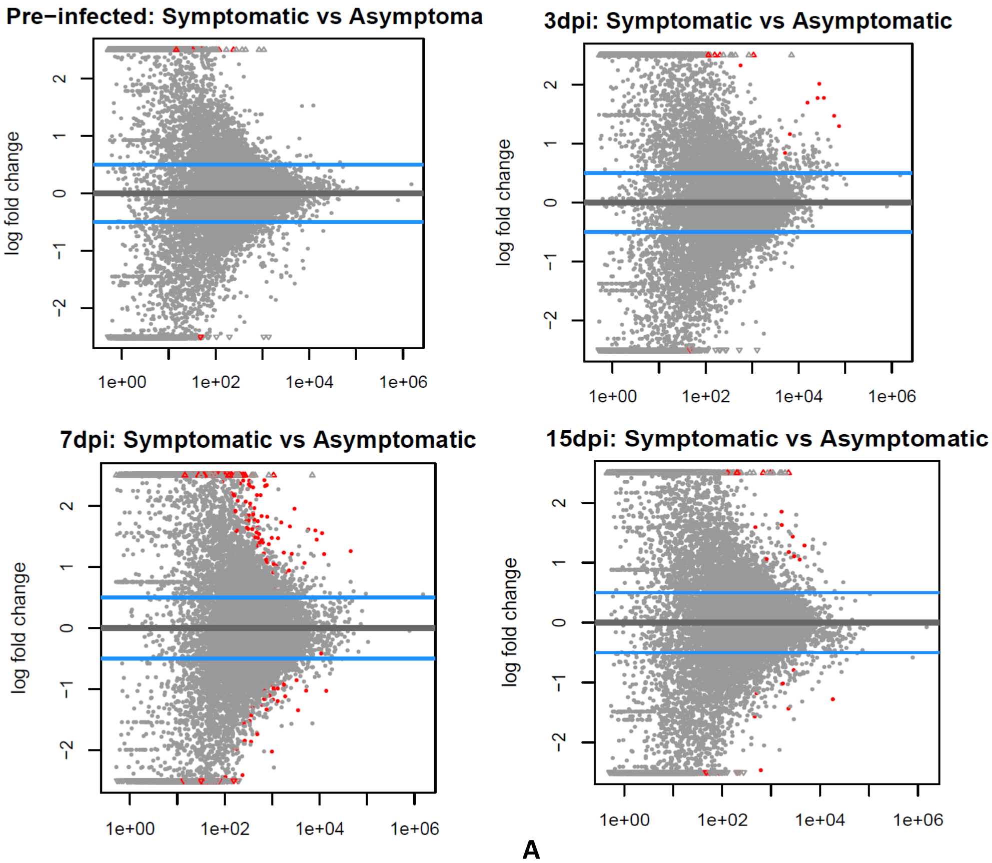
Pre−infected: Symptomatic vs Asymptoma
3dpi: Symptomatic vs Asymptomatic
7dpi: Symptomatic vs Asymptomatic
15dpi: Symptomatic vs Asymptomatic
log fold change
1e+00
1e+02
1e+04
1e+06

A

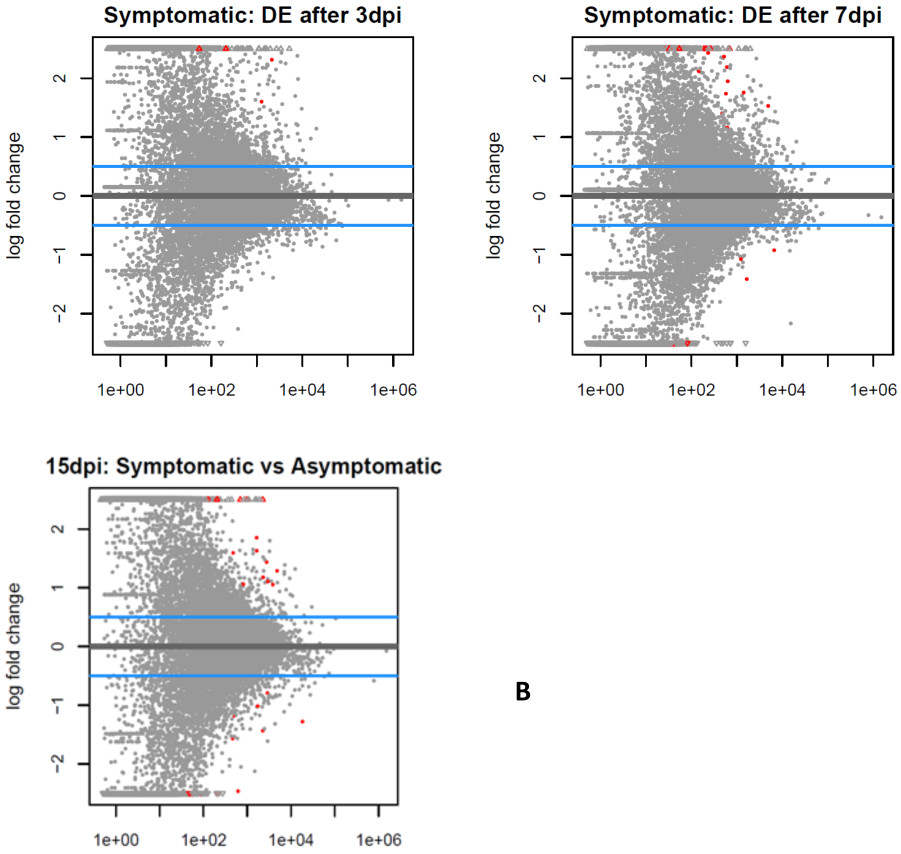
Symptomatic: DE after 3dpi
Symptomatic: DE after 7dpi
15dpi: Symptomatic vs Asymptomatic
log fold change
1e+00
1e+02
1e+04
1e+06

B
